# Lower BAFF Levels in Myasthenic Patients Treated with Glucocorticoids

**DOI:** 10.1007/s00005-021-00626-5

**Published:** 2021-08-02

**Authors:** Ewa Sobieszczuk, Piotr Szczudlik, Justyna Kubiszewska, Beata Szyluk, Marta Lipowska, Małgorzata Dutkiewicz, Anna Kostera-Pruszczyk

**Affiliations:** 1 Department of Neurology, Medical University of Warsaw, Banacha 1a, 02-097 Warsaw, Poland; 2 Department of Immunology, Biochemistry and Nutrition, Medical University of Warsaw, Warsaw, Poland

**Keywords:** Myasthenia gravis, BAFF, B-cell activating factor, Glucocorticoids, MG, Cytokine

## Abstract

B-cell activating factor (BAFF), a member of tumor necrosis factor family, activates B cells, promotes their survival and proliferation. BAFF is considered to have an influence on development of autoimmune diseases including myasthenia gravis (MG). We aimed to evaluate BAFF serum levels in MG patients, their potential connection with therapy and course of MG. Cross-sectional study. Two hundred eighteen adult patients with MG (67% women, age: 18–89 years, 82.6% AChR antibody seropositive (AChRAb(+)). Serum BAFF levels, their relationship with severity of clinical symptoms, therapy conducted, clinical and demographic features and other factors were analyzed. Patients with AChRAb(+) MG demonstrated significantly higher BAFF levels than MuSK-MG patients (831.2 ± 285.4 pg/ml vs. 745.6 ± 633.4 pg/ml, respectively; *p* = 0.030). Serum BAFF levels in women were significantly higher than in men (855.9 ± 302.5 vs. 756.6 ± 289.4, respectively; *p* = 0.017). Mean serum BAFF level was significantly decreased in patients who were ever treated with corticosteroids (CS) (770.4 ± 327.8 pg/ml vs. 891.3 ± 246.1 pg/ml, respectively; *p* = 0.001). Thymoma-MG patients demonstrated significantly lower BAFF levels (671.2 ± 244.9 vs. 833.5 ± 302.4, respectively; *p* = 0.044). Thymectomized patients did not differ in BAFF levels from the MG patients who had not undergone thymectomy. In multiple linear regression model, recent CS therapy and male sex were found to be independent predictors of lower BAFF levels. Serum BAFF level is decreased in patients treated with CS, which may suggest inhibiting influence of CS on BAFF—a potential mechanism contributing to the effectiveness of such therapy.

**Supplementary Information:**

The online version contains supplementary material available at 10.1007/s00005-021-00626-5.

## Introduction

B-cell activating factor (BAFF) belongs to tumor necrosis factor (TNF) family and is a crucial factor for development and survival of B lymphocytes. Several of its numerous roles include modifying of pro- and anti-apoptotic signals, initiating change for B class lymphocytes, inducing proliferation and secretion of T-cell response cytokines. Binding BAFF to its receptor on B cells results in stimulation of B cells and promotes antibody production by several different mechanisms, increasing B cells survival and proliferation also with blockade of self-reactive B cells. BAFF was confirmed to be one of key factors in promoting autoimmunity (Khan et al. 2013; Lahiri et al. 2012; Moore et al. 1999; Pillai et al. 2011). Its elevated serum levels have been demonstrated in myasthenia gravis (MG), lupus erythematosus, rheumatoid arthritis, Sjogren’s syndrome, autoimmune hepatitis, primary biliary cirrhosis and Graves’ disease (Chen et al. 2014; Kang et al. 2016; Kim et al. 2008; Migita et al. 2010; Vannucchi et al. 2012).

Myasthenia gravis is an autoimmune disorder of neuromuscular junction marked by skeletal muscle weakness and fatigability. In almost 90% of patients, it is caused by circulating antibodies against acetylcholine receptors (AChR); MuSK autoantibodies are present in approximately 5% of MG patients (Hoch et al. 2001; Lindstrom et al. 1976). MG can co-exist with various autoimmune diseases, most frequently (26.8%) with autoimmune thyroid diseases (Kubiszewska et al. 2016). It is typically treated with acetylcholinesterase inhibitors and immunosuppressants, and, in selected cases, with thymectomy. Higher serum BAFF levels in MG patients in comparison with healthy controls have been already reported (Kang et al. 2016; Kim et al. 2008; Ragheb et al. 2008; Scuderi et al. 2011). It prompted us to evaluate possible relationship of BAFF with the type of treatment and other factors within specific subgroups of MG patients.

## Patients and Methods

### Subjects

Two hundred eighteen adult patients with MG were enrolled in this study. Clinical diagnosis of MG was confirmed by positive result of repetitive nerve stimulation test or SFEMG and/or serum anti-AChR or anti-MuSK antibodies levels. 67% of tested patients were women; age ranged 18–89 years; 82.6% of subjects were seropositive for AChR antibodies (AChRAb(+)). 56.4% of patients were classified as early-onset MG (EOMG; ≤ 50 years), 37.2% as late-onset MG (LOMG; > 50 years); 6.4% had thymoma-MG (T-MG). Majority (56.4%) of the subjects have ever received corticosteroids (CS) therapy, 46.3% were treated with CS within last three months. 42.7% of all patients underwent thymectomy. The severity of symptoms was assessed with Myasthenia Gravis Foundation of America clinical classification scale (MGFA Classification). Results of treatment were assessed according to the MGFA Post-Intervention Status scale. Demographic, clinical and treatment of MG patients were summarized in Table 1.

**Table 1:** Demographic, clinical and treatment of MG patients

Variable	Value
Gender (number of patients)	
Male	72 (33%)
Female	146 (67%)
Current age (years)	
Mean ± SD	51.3 ± 18.7 years
Disease duration (years)	
Mean ± SD	9.2 ± 9.0 years
Age of onset (years)	
Mean ± SD	42.5 ± 22.0 years
Type of MG (number of patients)	
EOMG	123 (56.4%)
LOMG	81 (37.2%)
T-MG	14 (6.4%)
Serological status (number of patients)	
AChRAb(+)	180 (82.6%)
MuSK(+)	9 (4.1%)
AChRAb(–), MuSK(–)	29 (13.3%)
MGFA score (number of patients)	
0	46 (21.1%)
I	37 (17.0%)
II	112 (51.4%)
III	20 (9.2%)
IV	3 (1.4%)
Post-intervention status (number of patients)	
Remission	14 (6.4%)
Pharmacologic remission	42 (19.3%)
Improvement	123 (56.4%)
No improvement	20 (9.2%)
Worsening	16 (7.3%)
No data	3 (1.4%)
Treatment within last 3 months (number of patients)	
AChE inhibitors only	113 (51.8%)
Glucocorticoids	67 (30.7%)
Other immunosuppression	4 (1.8%)
IS + CS	34 (15.6%)
Glucocorticoids in the past (number of patients)	
Yes	123 (56.4%)
No	95 (43.6%)
Thymectomy (number of patients)	
Yes	93 (42.7%)
No	120 (55.0%)
No data	5 (2.3%)

### Methods

BAFF serum levels were measured in duplicate by ELISA (Human BAFF/BLyS/TNFSF13B Immunoassay, Quantikine ELISA, R&D Systems cat. no DBLYSOB) according to the manufacturer’s instruction.

### Statistical Analysis

All continuous data were expressed as means and standard deviations (SDs). To test distribution of continuous variables, we used Kolmogorov–Smirnov or Shapiro–Wilk tests according to the size of different subgroups. The Student’s *t* test and Mann–Whitney test were used to compare continuous variables between two groups as appropriate. Differences between more than two groups were tested using ANOVA with Bonferroni post hoc tests and Kruskal–Wallis test with post hoc multiple comparisons (all pairwise) as appropriate. Correlations were assessed using Pearson’s correlation coefficients or Spearman’s correlation coefficients according to the data distribution. To test interactions among variables, multivariate linear regression analysis was applied, including all variables from univariate models with the minimum significance level of 0.05. For the statistical analysis, SPSS version 20.0 was used.

## Results

**Fig. 1:**
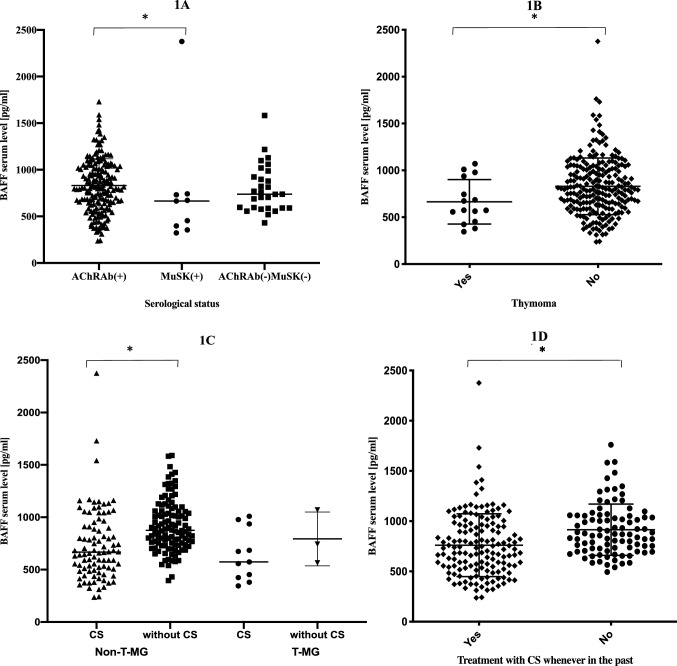
**A** Differences in BAFF serum levels depending on serological status of non-thymoma MG patients; **B** differences in BAFF serum levels between thymoma and non-thymoma MG patients; **C** differences in BAFF serum levels depending on treatment with CS within last three-month status in seropositive MG patient; **D** differences in BAFF serum levels depending on treatment with CS status in study cohort. *Non-T-MG* non-thymoma-MG, *T-MG* thymoma MG, *CS* corticosteroids; *p < 0.05

Patients with AChRAb(+) MG demonstrated significantly higher BAFF levels than MuSK-MG patients (831.2 ± 285.4 pg/ml vs. 745.6 ± 633.4 pg/ml, respectively; *p* = 0.030). Mean serum BAFF level was significantly decreased in patients who have ever received CS as compared with the remaining group (770.4 ± 327.8 pg/ml vs. 891.3 ± 246.1 pg/ml, respectively; *p* = 0.001). Serum BAFF levels in patients treated with CS within last three months were significantly decreased in comparison with those who have not received such therapy recently (723.8 ± 329.2 pg/ml vs. 914.6 ± 243.5 pg/ml, respectively; *p* < 0.001). We have performed analysis depending on serological status; for AChRAb(+) MG patients treated with CS within last three months, mean BAFF levels were 730.5 ± 295.8 vs. 930.7 ± 239.5 for untreated (*p* ≤ 0.001); for patients treated with CS whenever in the past, mean BAFF levels amounted 778.8 ± 301.0 vs. 907.8 ± 243.0 pg/ml for untreated (*p* = 0.002). We have also found such differences within AChRAb(–)MuSK(–) MG patients: those treated with CS within last three months as well as patients treated with CS whenever in the past showed significantly lower BAFF levels in comparison with untreated (613.97 ± 127.7 pg/ml vs. 855.4 ± 258.6 pg/ml, respectively; *p* = 0.009; 626.6 ± 147.9 vs. 873.9 ± 257.2 pg/ml, respectively; *p* = 0.006). Patients using other than CS immunosuppressants (IS) had no statistically significant difference in BAFF levels in comparison with patients without CS and/or other IS therapy (860.3 ± 274.7 pg/ml IS vs. 910.6 ± 245.6 pg/ml no IS; *p* = 0.481) There were also no differences between subgroups taking CS only vs. CS plus other IS (*p* = 0.468). BAFF did not correlate with AChRAb or anti-MuSK antibodies levels. T-MG patients demonstrated significantly lower BAFF levels than the non-T-MG (respectively 671.2 ± 244.9 vs. 833.5 ± 302.4 pg/ml; *p* = 0.044). There were also no differences in BAFF levels between T-MG patients treated with CS recently or in the past vs. untreated (*p* = 0.243). Thymectomy in the past had no influence on BAFF levels (*p* = 0.426). BAFF levels were significantly higher in women (855.9 ± 302.5 pg/ml vs. 756.6 ± 289.4, respectively; *p* = 0.017), but they were not correlated with the age of patient or the age of onset. Severity of MG symptoms considered as MGFA score was negatively correlated to the BAFF level (*p* = 0.005), but it was dependent on the use of CS within last three months. Within group of patients in remission, serum BAFF levels did not significantly differ from those with active form of disease (*p* = 0.14). There was no statistically significant difference in BAFF levels between EOMG and LOMG patients (*p* = 0.756); we also did not find any differences depending on MGFA Post-Intervention Status (*p* = 0.325). Results of comparison of BAFF levels between subgroups of MG patients are summarized in Table 2. In multivariate linear regression analysis, independent predictors of lower BAFF levels were: recent treatment of CS and male sex (Table 3). Figure 1A–D presents differences in BAFF levels depending on the serological status of non-T-MG patients (1A), presence of thymoma (1B), treatment with prednisone within last three months in T-MG vs. non-T-MG patients (1C) and treatment with CS in the past (1D). Detailed data on BAFF serum levels and MGFA status of patients are provided in supplementary material (Table S1).

**Table 2:** Results of comparison of BAFF levels between subgroups of MG patients

	BAFF (pg/ml)		Significance
	Mean	SD	
CS therapy within last 3 months			
Yes	723.8	329.2	< 0.001
No	914.6	243.5	
CS therapy in the past			
Yes	770.4	327.8	0.001
No	891.3	246.1	
Gender			
Women	855.9	302.5	0.017
Men	756.6	289.4	
Thymoma			
Yes	671.2	244.9	0.044
No	833.5	302.4	
Serological status			
AchRAb( +)	831.2	285.4	0.030
MuSK( +)	745.6	633.4

**Table 3:** Multivariate linear regression model

	Unstandardized coefficients		Standardized coefficients	*t*	Significance
	*B*	SE	Beta		
(Constant)	1127.500	82.943		13.594	0.000
Treatment with CS within last 3 months	–140.592	48.979	–0.233	–2.870	**0.005**
Thymoma-MG	–91.713	79.750	–0.075	–1.150	0.251
Treatment with CS in the past	–32.826	47.863	–0.054	–0.686	0.494
MGFA score	–37.668	20.394	–0.122	–1.847	0.066
Male gender	–87.661	41.815	–0.137	–2.096	**0.037**
*R* = 0.369; *R*^2^ = 0.136; Adjusted *R*^2^ = 0.116; *p* = 0.000					

*P*-values marked with bold indicate statistically significant* p*-values

Predictors of BAFF levels

## Discussion

Since its discovery in 1999 (Schneider et al. 1999), the role of BAFF in autoimmunity has been widely proved (Chen et al. 2014; Ferraccioli and Gremese 2017; Lahiri et al. 2012; Pillai et al. 2011). It has been reported that serum BAFF levels are increased in many autoimmune disorders, including MG (Kang et al. 2016; Kim et al. 2008; Migita et al. 2010; Ragheb et al. 2008; Scuderi et al. 2011; Thangarajh et al. 2006; Vannucchi et al. 2012). Mechanisms of BAFF have been described by several research groups (Chen et al. 2014; Hu et al. 2019; Kalled 2005; Khan et al. 2013; Pillai et al. 2011; Qin et al. 2011; Rauch et al. 2009; Schneider et al. 1999; Swee et al. 2010; Tang et al. 2018). This study has tested the potential connection between serum BAFF levels and laboratory or clinical features, type of MG, type of treatment and other factors.

We have observed significantly higher BAFF levels in patients with AChRAb(+) MG in comparison with MuSK-MG, although, as in previous reports, our MuSK-MG group was small (Guptill et al. 2015; Ragheb et al. 2008). It was already described that AChRAb(+) MG patients have BAFF levels higher than healthy controls (Kang et al. 2016). Also MuSK-MG patients were reported to have higher BAFF levels than healthy individuals (Guptill et al. 2015), while to our knowledge, there were no differences between AChRAb(+) and MuSK-MG patients described as seen in our material. Trend towards higher BAFF levels in AChRAb(+) MG patients was observed in Ragheb et al. (2008) and Kim et al. (2008) studies, but it did not reach the statistical significance, possibly due to the sample size.

We have demonstrated significant decrease in BAFF levels in patients treated with CS within last three months; this relationship was seen also when CS were used at any time in the past in comparison with patients never treated with CS. Not only AChRAb(+), but also AChRAb(–)MuSK(–) MG patients had lower BAFF levels when treated with CS therapy. Only one MuSK-MG patient was not treated with CS, though we were not able to perform proper analysis within this subgroup. Lowering BAFF as the possible result of CS therapy has been already observed and reported in Kang et al. (2016) and Scuderi et al. (2011) studies, but Kim et al. (2008) did not demonstrate such differences. All previous studies reported the influence of current CS use only and did not describe differences in subgroups depending on serological status. Although we demonstrated that BAFF levels are higher in AChRAb(+) than MuSK-MG, we were not able to evaluate if there were any differences in BAFF levels depending on CS treatment status in MuSK-MG patients, as their number was small. Our data confirm that BAFF is decreased in patients using CS; the question remains how long-lasting this effect is, and whether CS’s potential influence on BAFF level parallels changes in clinical status of the patient.

We did not see differences in BAFF levels depending on any IS treatment other than CS. This could indicate that various pathways are responsible for efficacy of IS than CS in MG.

Similarly to Ragheb et al. (2008), we did not find any correlation between BAFF and AChRAb levels. Such relationship was observed by Kang et al. (2016) in 20 AChRAb(+) MG patients. None of previous studies tested correlation between BAFF and AChRAb levels in both immuno-suppressed and non-immuno-suppressed patients separately. Ragheb et al. (2008) included only non-immuno-suppressed patients.

Our study demonstrated significantly higher BAFF levels in female MG patients than in males. Similar trend was reported in Ragheb et al.(2008), while Kang et al. (2016) observed no differences between males and females in BAFF levels, in both studies, the M:F groups were small. There are few data about gender differences in BAFF levels in autoimmune diseases other than MG, although Panchanathan and Choubey (2013) reported that levels of the BAFF mRNA were measurably higher in cells isolated from females than male mouse models of lupus diseases and murine BAFF expression was found to be up-regulated by estrogen and interferons. In recent MG study by Deng et al. (2019), polymorphism in BAFF gene was also found to be gender-dependent: frequency of genotype AA in female MG patients was significantly elevated compared to control group. However, some studies showed no significant differences in BAFF levels between women and men with autoimmune diseases (Lin et al. 2016; Mameli et al. 2016).

Our thymoma patients had significantly lower BAFF levels than non-T-MG. T-MG patients, contrarily to the patients without thymoma, did not differ significantly in BAFF levels depending on CS therapy status. Kim et al. (2008) reported trend to higher BAFF levels in thymoma or thymic hyperplasia patients, but the observation did not reach the statistical significance. None of the other studies reported significant differences in BAFF levels between T-MG patients and non-T-MG. We have not found differences in BAFF levels depending on the history of thymectomy. This observation is consistent with Kim et al. (2008) and Scuderi et al. (2011) studies.

Although we have observed a faint negative correlation between BAFF levels and MGFA score, only recent CS treatment and male gender were found to be independent predictors of lower BAFF levels. Consistently, in several studies, there were no differences in BAFF levels related to the severity of symptoms (Guptill et al. 2015; Kang et al. 2016; Kim et al. 2008; Ragheb et al. 2008; Scuderi et al. 2011).

In conclusion, our study confirms lower BAFF serum levels in AChRAb( +) but also AChRAb(–)MuSK(–) MG patients treated with CS—the question whether this effect is long-term and reflected in clinical status of patient remains open. The results of studies concerning BAFF as potential target for novel drugs in MG are contradictory. Recent experimental report showed dose-dependent, immunomodulatory distant effect resulting from BAFF receptor-specific mAb-siRNA-conjugate treatment in an in vivo model of MG (Ibtehaj and Huda 2017). Though, the result of belimumab (monoclonal antibody against BAFF) study in MG, already approved for treating SLE, was negative (Dalakas 2019; Hewett et al. 2018). Therefore, we conclude that BAFF inhibition with novel drugs could be promising (Dalakas 2019; Huang et al. 2018; Nakayamada and Tanaka 2016), yet not well-established pathway in autoimmune diseases possibly including also myasthenia gravis.

## Supplementary Information

Below is the link to the electronic supplementary material.

Supplementary file1 (DOC 301 KB)

## Data Availability

Yes.
