# Supplementary material for: Lower BAFF Levels in Myasthenic Patients Treated with Glucocorticoids
Source: Arch Immunol Ther Exp (Warsz). 2021 Aug 2;69(1):22. doi: 10.1007/s00005-021-00626-5 (PMC8328853; doi:10.1007/s00005-021-00626-5)
Supplement: Supplementary file 1 — Supplementary file1 (DOC 301 KB) [file 5_2021_626_MOESM1_ESM.doc]

| **Table S1.** | |
| --- | --- |
| **BAFF (pg/ml)** | **MGFA score** |
| 1270 | No symptoms |
| 629 | No symptoms |
| 697 | No symptoms |
| 585 | No symptoms |
| 1206 | No symptoms |
| 965 | No symptoms |
| 382 | No symptoms |
| 395 | No symptoms |
| 799 | No symptoms |
| 1590 | No symptoms |
| 1029 | No symptoms |
| 772 | No symptoms |
| 873 | No symptoms |
| 870 | No symptoms |
| 951 | No symptoms |
| 484 | No symptoms |
| 887 | No symptoms |
| 1003 | No symptoms |
| 1321 | No symptoms |
| 729 | No symptoms |
| 1348 | No symptoms |
| 495 | No symptoms |
| 1411 | No symptoms |
| 1483 | No symptoms |
| 1324 | No symptoms |
| 1051 | No symptoms |
| 869 | No symptoms |
| 789 | No symptoms |
| 543 | No symptoms |
| 857 | No symptoms |
| 355 | No symptoms |
| 671 | No symptoms |
| 1583 | No symptoms |
| 590 | No symptoms |
| 555 | No symptoms |
| 577 | No symptoms |
| 1059 | No symptoms |
| 1218 | No symptoms |
| 555 | No symptoms |
| 703 | No symptoms |
| 978 | No symptoms |
| 1071 | No symptoms |
| 744 | No symptoms |
| 557 | No symptoms |
| 918 | I |
| 822 | I |
| 1145 | I |
| 523 | I |
| 1541 | I |
| 806 | I |
| 622 | I |
| 685 | I |
| 1142 | I |
| 768 | I |
| 664 | I |
| 839 | I |
| 754 | I |
| 922 | I |
| 440 | I |
| 728 | I |
| 965 | I |
| 548 | I |
| 891 | I |
| 533 | I |
| 640 | I |
| 888 | I |
| 1428 | I |
| 1043 | I |
| 1062 | I |
| 799 | I |
| 734 | I |
| 1099 | I |
| 1161 | I |
| 736 | I |
| 1098 | I |
| 1128 | I |
| 597 | I |
| 822 | I |
| 770 | I |
| 937 | I |
| 1010 | I |
| 484 | IIa |
| 668 | IIa |
| 373 | IIa |
| 237 | IIa |
| 946 | IIa |
| 1048 | IIa |
| 1084 | IIa |
| 955 | IIa |
| 1385 | IIa |
| 791 | IIa |
| 932 | IIa |
| 911 | IIa |
| 1067 | IIa |
| 1170 | IIa |
| 1208 | IIa |
| 1134 | IIa |
| 1054 | IIa |
| 1038 | IIa |
| 1269 | IIa |
| 832 | IIa |
| 372 | IIa |
| 1165 | IIa |
| 802 | IIa |
| 704 | IIa |
| 655 | IIa |
| 800 | IIa |
| 494 | IIa |
| 749 | IIa |
| 687 | IIa |
| 868 | IIa |
| 1191 | IIa |
| 935 | IIa |
| 1107 | IIa |
| 952 | IIa |
| 1018 | IIa |
| 1013 | IIa |
| 754 | IIa |
| 889 | IIa |
| 741 | IIa |
| 2376 | IIa |
| 430 | IIa |
| 687 | IIa |
| 738 | IIa |
| 594 | IIa |
| 878 | IIa |
| 762 | IIa |
| 988 | IIa |
| 424 | IIa |
| 631 | IIb |
| 909 | IIb |
| 791 | IIb |
| 800 | IIb |
| 855 | IIb |
| 436 | IIb |
| 682 | IIb |
| 590 | IIb |
| 1150 | IIb |
| 555 | IIb |
| 656 | IIb |
| 540 | IIb |
| 311 | IIb |
| 780 | IIb |
| 1314 | IIb |
| 590 | IIb |
| 1005 | IIb |
| 1058 | IIb |
| 586 | IIb |
| 643 | IIb |
| 804 | IIb |
| 1013 | IIb |
| 754 | IIb |
| 674 | IIb |
| 1054 | IIb |
| 784 | IIb |
| 651 | IIb |
| 903 | IIb |
| 781 | IIb |
| 1099 | IIb |
| 796 | IIb |
| 380 | IIb |
| 722 | IIb |
| 720 | IIb |
| 472 | IIb |
| 659 | IIb |
| 536 | IIb |
| 822 | IIb |
| 985 | IIb |
| 817 | IIb |
| 333 | IIb |
| 512 | IIb |
| 413 | IIb |
| 416 | IIb |
| 1730 | IIb |
| 1043 | IIb |
| 374 | IIb |
| 1089 | IIb |
| 397 | IIb |
| 453 | IIb |
| 732 | IIb |
| 814 | IIb |
| 518 | IIb |
| 897 | IIb |
| 590 | IIb |
| 1019 | IIb |
| 586 | IIb |
| 922 | IIb |
| 674 | IIb |
| 453 | IIb |
| 565 | IIb |
| 345 | IIb |
| 1127 | III |
| 835 | III |
| 645 | III |
| 1037 | III |
| 862 | III |
| 506 | III |
| 1159 | III |
| 442 | III |
| 1008 | III |
| 1295 | III |
| 813 | III |
| 698 | III |
| 1093 | III |
| 597 | III |
| 671 | III |
| 322 | III |
| 664 | III |
| 705 | III |
| 380 | III |
| 573 | III |
| 242 | IVb |
| 682 | IVb |
| 685 | IVb |
